# Towards an international taxonomy of integrated primary care: a Delphi consensus approach

**DOI:** 10.1186/s12875-015-0278-x

**Published:** 2015-05-22

**Authors:** Pim P. Valentijn, Hubertus J. M. Vrijhoef, Dirk Ruwaard, Inge Boesveld, Rosa Y. Arends, Marc A. Bruijnzeels

**Affiliations:** Jan van Es Institute, Netherlands Expert Centre Integrated Primary Care, Wisselweg 33, CB 1314 Almere, The Netherlands; Scientific Centre for Care and Welfare (Tranzo), Tilburg University, Tilburg, The Netherlands; Saw Swee Hock School of Public Health, National University of Singapore, Singapore, Singapore; Department of Patient and Care, University Hospital Maastricht, Maastricht, The Netherlands; Department of Health Services Research, School for Public Health and Primary Care, Faculty of Health, Medicine and Life Sciences, Maastricht University, Maastricht, The Netherlands; Department of Psychology, Health & Technology, University of Twente, Enschede, The Netherlands

**Keywords:** Integrated care, Primary care, Coordinated care, Delphi study, Taxonomy, Classification, Delivery of health care

## Abstract

**Background:**

Developing integrated service models in a primary care setting is considered an essential strategy for establishing a sustainable and affordable health care system. The Rainbow Model of Integrated Care (RMIC) describes the theoretical foundations of integrated primary care. The aim of this study is to refine the RMIC by developing a consensus-based taxonomy of key features.

**Methods:**

First, the appropriateness of previously identified key features was retested by conducting an international Delphi study that was built on the results of a previous national Delphi study. Second, categorisation of the features among the RMIC integrated care domains was assessed in a second international Delphi study. Finally, a taxonomy was constructed by the researchers based on the results of the three Delphi studies.

**Results:**

The final taxonomy consists of 21 key features distributed over eight integration domains which are organised into three main categories: *scope* (person-focused vs. population-based), *type* (clinical, professional, organisational and system) and *enablers* (functional vs. normative) of an integrated primary care service model.

**Conclusions:**

The taxonomy provides a crucial differentiation that clarifies and supports implementation, policy formulation and research regarding the organisation of integrated primary care. Further research is needed to develop instruments based on the taxonomy that can reveal the realm of integrated primary care in practice.

## Background

Developing integrated service delivery in a primary care setting is considered an important strategy to establish a more sustainable and affordable health care system [1, 2]. Despite the increasing popularity of organising integrated service models, a solid scholarly exploration of the concept of integrated primary care is limited [3]. Throughout this paper we refer to *integrated primary care* as ambulatory care settings in which a network of multiple professionals and organisations across the health and social care system provide accessible, comprehensive, and coordinated services to a population in a community. Existing integrated care models lack a primary care perspective that is based on an encompassing inter-sectorial system approach with a distinct community and socio-political focus [3, 4]. Consequently, there is a need to develop a common terminology and typology for integrated primary care in order to facilitate program implementation, policy formulation and research.

In a previous publication, we introduced the Rainbow Model of Integrated Care (RMIC) as a guide to understanding the complex and comprehensive nature of integrated primary care [3]. The model distinguishes six domains of integrated care (clinical, professional, organisational, system, functional and normative integration) and two primary care guiding principles (person-focused and population-based). The model is considered useful for understanding the complexity of integrated service delivery as a whole [5]. Based on these theoretical foundations of the RMIC, a draft taxonomy was developed that specified underlying key features of the six integrated care domains [6].

In our previous research, we conducted a Delphi study among a panel of experts from The Netherlands in order to investigate the appropriateness of the key features to achieve integrated primary care. The results of this Delphi study indicated that further work was needed to establish a common operational consensus regarding our taxonomy. The purpose of the present study is to further refine our taxonomy by testing it against international expert opinions in the field of integrated primary care. We aim to contribute to the ongoing debate of defining and specifying integrated care by addressing the following objectives:

1. Investigate the appropriateness of the key features to achieve integrated primary care among a panel of international experts.
2. Test the categorisation of the key features across the domains of the RMIC against international expert opinions.
3. Develop a consensus-based taxonomy derived from the results of the previous and present studies.

## Methods

**Fig. 1:**
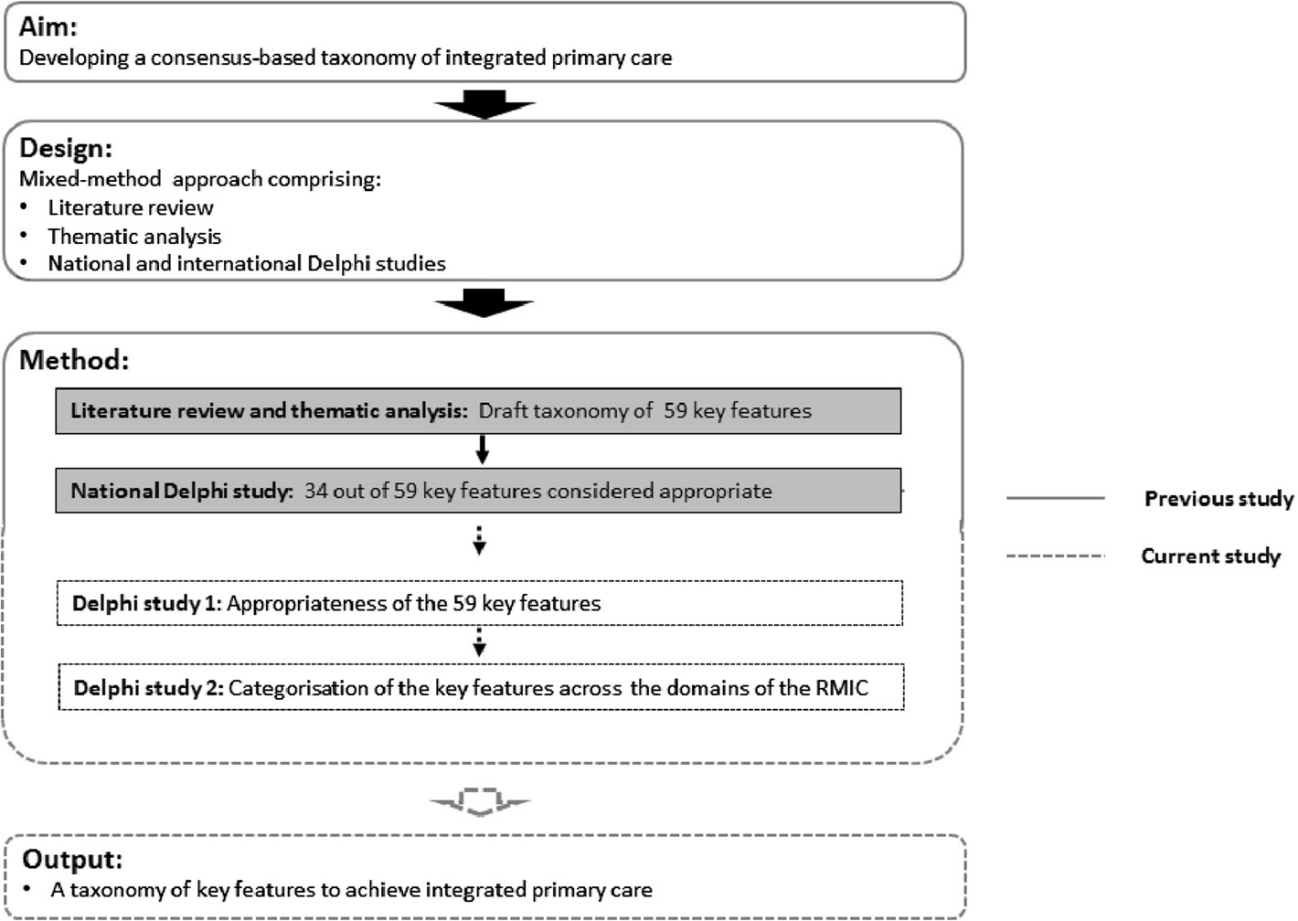
Study design

In the previous study, we developed a draft taxonomy of 59 key features based on a literature review and a thematic analyses method [6]. We performed a national Delphi study as a first step to deriving an operational consensus about our taxonomy. Continuing this line of research, for this current study, we developed two international Delphi studies to investigate the appropriateness of the taxonomy to achieve integrated primary care (see Fig. 1). First, the appropriateness of the original 59 key features was assessed by a panel of international experts. Second, another panel of international experts assessed the categorisation of the key features and their distribution across the domains of the RMIC. Finally, a consensus-based taxonomy was developed by using the results of all three Delphi studies (the previous Delphi study and the international Delphi studies presented in this article).

### Delphi consensus process

Two separate modified Delphi studies were conducted to: 1) investigate the appropriateness of the key features and, 2) test the categorisation across the domains of the RMIC. The modified Delphi methodology is a research technique designed to obtain opinions from experts through the use of a self-administrated questionnaire (Round 1) and of a physical meeting of experts (Round 2) to discuss the ratings of Round 1 [7]. Given the polymorphous nature of integrated care [4], the physical meetings enabled the experts to clarify each other’s perspectives on integrated care [8].

### Selection of participants

For each international Delphi study, a purposive sampling strategy was used to identify experts with experience in practice, science or policy in the deployment of integrated service models in a primary care setting. Potential experts were nominated using the following criteria: 1) scientific (performing research) experience and/or 2) practical (working in a professional or service organisation) experience regarding the organisation of integrated primary care. We attempted to balance the number of potential experts from each category in order to ensure each was represented in both studies. We did not include experts with an explicit policy or insurance background or patients in order to minimalize conflict of interest during the face-to-face meeting of the second Delphi round [6]. For example, the presence of policymaker or health insures could influence the (strategic) behaviour of the practice experts, as they are (financially) dependent of these stakeholders for the continuity of their practices. Experts who met the selection criteria were e-mailed an invitation with information on the research aims and details of the Delphi study. Only those who agreed to participate in both the initial online self-administrated questionnaire and the second face-to-face meeting were included. Experts who already participated within one of the previous Delphi studies were excluded.

### Delphi study 1

The first international Delphi study was conducted to test the appropriateness of the 59 features at an international level in order to augment the research we conducted on the appropriateness of the features in the previous national Delphi study. Thirty-seven international experts from an a priori list of participants of the World Congress on Integrated Care (2013) in Singapore [9] were approached by e-mail and invited to participate. During Round 1, the experts received a link to an online questionnaire. They were asked to rate the appropriateness of each feature to achieve integrated primary care on a nine-point Likert-scale, ranging from 1 (completely irrelevant) to 9 (extremely relevant). Experts were asked to comment on any of the features, to suggest possible rephrasing and to highlight any features that may have been missed in the initial list. Two e-mail reminders were sent to non-responders.

In Round 2, a face-to-face meeting of the expert panel took place during the World Congress on Integrated Care. The meeting was chaired by one member of the research team (HV). The goal of the meeting was to discuss the results of Round 1 and, after the discussion, to reassess the value of the appropriateness of the features to achieve integrated primary care. Based on the results of Round 1, a summary report was provided to the experts at the meeting with the following key feedback information for each feature: 1) respondents’ own ratings, 2) median agreement rating, 3) summary of qualitative comments, and 4) whether a consensus was achieved at Round 1. Because of time, during the second round, we decided to only discuss the features that were not considered appropriate in the first round.

To begin the discussion over a disputed feature value, panel members who had rated the disputed feature with either its highest and lowest scores were asked to clarify their considerations. Next, a short discussion among all group members took place to explore if differences were due to real disagreement or to a misunderstanding or misinterpretation of the feature [8]. Finally, the experts were asked to individually rate the feature once again on their summary report. During the discussion, notes were taken by two observers.

### Delphi study 2

A second international Delphi study was conducted to: 1) refine the descriptions of the domains of the RMIC and 2) categorise the features under one of the domains of the RMIC. Only key features that were considered appropriate within one of the previous Delphi studies (national and international) were included in the final Delphi study. Thirty-six experts from an a priori list of participants at the 14^th^ International Conference on Integrated Care (2014) in Brussels [9, 10] were invited by e-mail. In Round 1, the experts received a link to an online questionnaire and were asked to categorise each feature into one of the domains of the taxonomy: 1) clinical integration, 2) professional integration, 3) organisational integration, 4) system integration, 5) functional integration, 6) normative integration, and 7) person-focused and population-based care. Two e-mail reminders were sent to non-responders.

In the second round, a face-to-face expert panel meeting took place. A member of the research team (MB) chaired this meeting and facilitated the panel discussion. Based on the results of Round 1, a summary report was provided to the experts with the following key feedback information: 1) respondents’ own categorisation of each feature, 2) whether consensus was achieved regarding the categorisation in Round 1, and 3) a summary of qualitative comments. First, an iterative group discussion was conducted about the descriptions of the seven domains of the draft taxonomy. Second, the features that did not result in a consensus regarding their categorisation were discussed. Next, a short discussion among all group members took place. Finally, the experts were asked to once again individually categorise the features on which no consensus was reached in the first round. During the discussion, notes were taken by two observers.

### Synthesis of the results

**Fig. 2:**
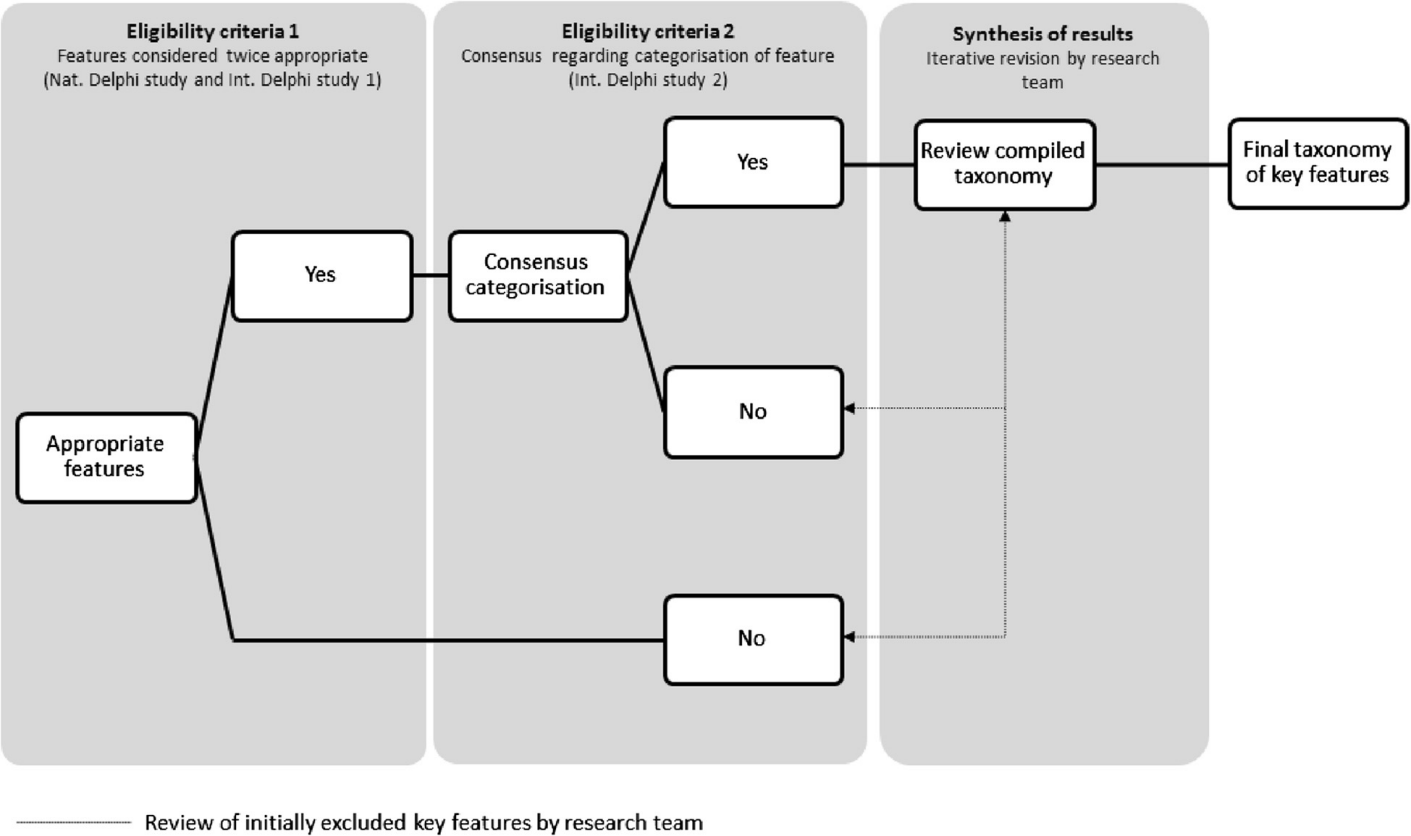
Flowchart of the synthesis of results. Steps and criteria used to construct final taxonomy of key features

Based on the results of the previous and current Delphi studies, a final version of the taxonomy was constructed. The research team synthesised the results and comments provided by the experts to produce a final taxonomy of features. To be initially included, features had to meet the following sequential eligibility criteria: 1) features had to be considered appropriate in Delphi study 1 as well as the national Delphi study and 2) a consensus had to be reached regarding categorisation based on the results of Delphi study 2 (see Fig. 2). Thereafter, three authors (PV, MB and IB) independently assessed the compiled taxonomy of features. To ensure a comprehensive analysis, the authors checked if each domain contained sufficient key features and iterative revisions were made. Features that were identical or nearly identical were aggregated. All authors gave feedback on the final taxonomy to refine the descriptions of the key features. PV summarised the feedback and the taxonomy accordingly.

### Data analysis

Criteria of the RAND UCLA appropriateness method were used to analyse the data from the previous national Delphi study and the current Delphi study 1 [8]. We categorized the overall panel median as follows: 1–3 as inappropriate, 4–6 as equivocal and 7–9 as appropriate. Agreement signified that ≥ 70 % of panellists’ ratings were within the same 3-point region (that is, 1–3, 4–6, or 7–9) as the observed median. A feature was defined as “appropriate” with an overall panel median score of ≥ 7 and a level of agreement of ≥ 70 % within the 3-point region 7–9. A panel median of 4–6 or median with consensus of ≤ 70 % within the same 3-point region was defined as “equivocal”. A feature with a panel median of 1–3 and a level of agreement of ≥ 70 % within the 3-point region 1–3, was defined as “inappropriate”. The decision rules used in the national Delphi study and international Delphi study 1 are shown in Table 1. During Delphi study 1, PV and HV tabulated and discussed qualitative responses after Round 1, and circulated the results to the experts for Round 2. For international Delphi study 2, agreement signified that ≥ 60 % of panellists categorised a feature within the same domain. During Delphi study 2, PV and MB tabulated and discussed qualitative responses after Round 1 and circulated the results to the participants for Round 2. Quantitative analysis was done using Statistical Package for the Social Sciences (SPSS) version 21 for Windows (IBM Statistics).

**Table 1:** Decision rules national and international Delphi study 1

		Median (1–3)	Median (4–6)	Median (7–9)
Round 1	Agreement (≤70 %)	Equivocal; discussion Round 2	Equivocal; discussion Round 2	Equivocal: discussion Round 2
	Agreement (≥70 %)	Inappropriate; excluded after Round 1	Equivocal; discussion Round 2	Appropriate; included after Round 1
Round 2	Agreement (≤70 %)	Equivocal	Equivocal	Equivocal
	Agreement (≥70 %)	Inappropriate	Equivocal	Appropriate

### Ethics

This study has conformed to the principles embodied in the Declaration of Helsinki. All experts approved their participation by an informed consent. As this study does not involve patients or study subjects, according to the Dutch Medical Research in Human Subjects Act (WMO), this is exempt from ethical approval in The Netherlands.

## Results

For Delphi study 1, we asked 37 experts to participate; 16 were willing to participate, 16 completed Round 1 and 15 completed Round 2. Participants had experience with integrated care in 11 different countries (Australia, Austria, Belgium, El Salvador, Russia, Singapore, Spain, Sweden, The Netherlands, UK and USA). For Delphi study 2, 36 people were invited. Eight experts completed both rating rounds. The experts gained their experience with integrated care in five different countries (Australia, Belgium, Germany, New Zealand and The Netherlands). The main reason reported by experts for not participating in the international Delphi studies 1 or 2 was their lack of availability for the second round face-to-face meeting. Table 2 describes the characteristics of the participants of the two Delphi studies.

**Table 2:** Participants’ characteristics of the two international Delphi studies

	Delphi study 1		Delphi study 2	
	Round 1	Round 2	Round 1	Round 2
Number of participants	16	15	8	8
Dominant background, n (%)				
Practical	7 (44)	6 (40)	4 (50)	4 (50)
Scientific	9 (56)	9 (60)	4 (50)	4 (50)
Years of experience, mean (SD), range	9.5 (6.7), 3–25	9.5 (6.9), 3–25	13.4 (8.6), 4–25	13.4 (8.6), 4-25
<5	2 (12)	2 (13)	2 (25)	2 (25)
5–10	10 (63)	9 (60)	2 (25)	2 (25)
>10	4 (25)	4 (27)	4 (50)	4 (50)
Experience gained in country, n				
Australia	1	-	2	2
Austria	1	1	-	-
Belgium	2	2	1	1
El Salvador	1	1	-	-
Germany	-	-	1	1
New Zealand	-	-	2	2
Russia	1	1	-	-
Singapore	5	5	-	-
Spain	1	1	-	-
Sweden	1	1	-	-
The Netherlands	1	1	2	2
United Kingdom	1	1	-	-
United States of America	1	1	-	-

### Delphi study 1

After the first round, the international experts of Delphi study 1 considered 25 of the 59 proposed features of the taxonomy appropriate. (The overall panel median was between 7 and 9 with a consensus ≥ 70 % within this 3-point region, see Table 3, columns 3 and 4). Thirty-four features were rated as equivocal (overall panel median between 4 and 6 or median with consensus ≤ 70 % within the same 3-point region). Furthermore, the experts suggested five new features during this round (see Table 3, column 1): incentive systems (no. 43), community participation (no. 44), universal health coverage (no. 45), single point of access (no. 46) and alignment of regulatory frameworks (no. 47). After reviewing the results, the research team initially categorised the newly added features within the domain of system integration of the draft taxonomy. This addition led to a list of 64 key features.

**Table 3:** Results of the national Delphi and international Delphi study 1 and 2

Initial taxonomy of key features	Identification appropriateness and categorisation of key features									
Literature review and thematic analysis	National Delphi study	Delphi study 1					Delphi study 2			
		Round 1 (*n* = 16)		Round 2 (*n* = 15)			Round 1 (*n* = 8)	Round 2 (*n* = 8)		
Initial taxonomy of key features^#^	Final consensus^#^	Panel median (30th and 70th percentile)	Agreed (%)	Panel median (30th and 70th percentile)	Agreed (%)	Final consensus	Categorisation	Categorisation	Agreed (%)	Final consensus
Clinical integration										
1. Centrality of client needs	Appropriate	8 (8 − 8.9)	93.8	N/A	N/A	Appropriate	PP		75	Yes
2. Case management	Appropriate	8 (7.1 − 8)	75	N/A	N/A	Appropriate	CI		62.5	Yes
3. Patient education	Equivocal	7 (6 − 8)	62.5	8 (7 − 8.2)	80	Appropriate	PP		75	Yes
4. Client satisfaction	Equivocal	8 (7.1 − 8)	87.5	N/A	N/A	Appropriate	PP		62.5	Yes
5. Continuity	Appropriate	8.5 (8 − 9)	93.8	N/A	N/A	Appropriate	CI		62.5	Yes
6. Interaction between professional and client	Appropriate	7 (6.1 − 7.9)	68.8	7 (7 − 8)	86.7	Appropriate		CI	62.5	Yes
7. Individual multidisciplinary care plan	Appropriate	8 (7 − 8.9)	93.8	N/A	N/A	Appropriate	CI		62.5	Yes
8. Information provision to clients	Equivocal	6.5 (5 − 7)	50	7 (5.8 − 8)	66.7	Equivocal	N/A	N/A	N/A	N/A
9. Comprehensive care services^aa^	Appropriate	7 (6 − 8)	62.5	8 (7 − 9)	80	Appropriate			DA	No
10. Client participation	Equivocal	8 (7 − 8)	75	N/A	N/A	Appropriate	PP		62.5	Yes
11. Population needs	Appropriate	8 (7.1 − 9)	81.3	N/A	N/A	Appropriate	PP		87.5	Yes
12. Self-management^a^	Equivocal	7 (6.1 − 7.9)	68.8	8 (7 − 8)	93.3	Appropriate	PP		75	Yes
Professional integration										
13. Inter-professional education	Appropriate	7 (6 − 7.9)	62.5	7 (6.8 − 8)	73.3	Appropriate		PI	62.5	Yes
14. Shared vision between professionals	Appropriate	8 (8 − 9)	93.8	N/A	N/A	Appropriate		NI	75	Yes
15. Agreements on interdisciplinary collaboration	Appropriate	7 (7 − 7.9)	75	N/A	N/A	Appropriate		PI	87.2	Yes
16. Multidisciplinary guidelines and protocols	Appropriate	7.5 (7 − 8)	75	N/A	N/A	Appropriate			DA	No
17. Inter-professional governance	Appropriate	7 (7 − 7.9)	75	N/A	N/A	Appropriate		OI	62.5	Yes
18. Interpersonal characteristics^a^	Equivocal	7 (6 − 7.9)	56.3	7 (6 − 7)	53.3	Equivocal	N/A	N/A	N/A	N/A
19. Professional leadership^a^	Equivocal	7.5 (6.1 − 8)	68.8	8 (6.8 − 9)	73.3	Appropriate			DA	No
20. Environmental awareness	Equivocal	5 (5 − 6)	75	6 (5 − 7)	53.3	Equivocal	N/A	N/A	N/A	N/A
21. Value creation for the professional	Appropriate	7 (6 − 7)	62.5	8 (7 − 9)	100	Appropriate	PI		75	Yes
22. Performance management	Equivocal	7 (7 − 8)	75	N/A	N/A	Appropriate		PP	87.5	Yes
23. Creating interdependence between professionals^a^	Equivocal	7 (6 − 8)	62.5	7 (7 − 8)	86.7	Appropriate	PI		62.5	Yes
Organisational integration										
24. Value creation for organisation	Equivocal	8 (7 − 8)	75	N/A	N/A	Appropriate			DA	No
25. Inter-organisational governance	Appropriate	8 (8 − 9)	81.3	N/A	N/A	Appropriate	OI		75	Yes
26. Informal managerial network	Equivocal	6 (6 − 7)	43.8	5 (3 − 6)	53.3	Equivocal	N/A	N/A	N/A	N/A
27. Interest management^a^	Appropriate	7 (6.1 − 7.9)	68.8	7 (7 − 7.2)	86.7	Appropriate			DA	No
28. Performance management^a^	Appropriate	7 (6 − 7)	62.5	6 (6 − 7)	46.7	Equivocal	OI		62.5	Yes
29. Population needs as binding agent	Appropriate	8 (7 − 8)	75	N/A	N/A	Appropriate	PP		75	Yes
30. Organisational features^a^	Equivocal	6.5 (5.1 − 7)	31.3	6 (4 − 7)	46.7	Equivocal	N/A	N/A	N/A	N/A
31. Inter-organisational strategy	Appropriate	7.5 (7 − 8)	75	N/A	N/A	Appropriate	OI		62.5	Yes
32. Managerial leadership	Appropriate	7 (6 − 8)	62.5	8 (7 − 9)	86.7	Appropriate			DA	No
33. Learning organisations	Appropriate	7.5 (7 − 8)	81.3	N/A	N/A	Appropriate		FI	62.5	Yes
34. Co-location policy^a^	Equivocal	5.5 (5 − 6)	62.5	6 (4.8 − 7)	40	Equivocal	N/A	N/A	N/A	N/A
35. Skills management^a^	Appropriate	7 (6 − 7)	56.3	7 (6 − 7)	66.7	Equivocal	PI		62.5	Yes
36. Creating interdependence between organisations^a^	Equivocal	7 (6 − 8)	56.3	7 (7 − 8)	80	Appropriate	OI		62.5	Yes
System integration										
37. Social value creation^a^	Equivocal	8 (7 − 8)	75	N/A	N/A	Appropriate		PP	75	Yes
38. Available resources	Equivocal	6 (5 − 7)	43.8	6 (4.8 − 7)	53.3	Equivocal	N/A	N/A	N/A	N/A
39. Population features	Equivocal	6 (4.1 − 7)	31.3	7 (5.6 − 8)	60	Equivocal	N/A	N/A	N/A	N/A
40. Stakeholder management	Appropriate	8 (7 − 8.9)	87.5	N/A	N/A	Appropriate			DA	No
41. Good governance	Equivocal	7 (6.1 − 7)	68.8	7 (7 − 8)	86.7	Appropriate			DA	No
42. Environmental climate	Equivocal	6 (6 − 7)	50	7 (7 − 8.2)	80	Appropriate	SI		75	Yes
43. Incentive systems^b^	N/A	N/A	N/A	8 (8 − 9)	93.3	Appropriate		FI	87.5	Yes
44. Community participation^b^	N/A	N/A	N/A	8 (7 − 8)	93.3	Appropriate			DA	No
45. Universal health coverage^b^	N/A	N/A	N/A	7 (5 − 7)	66.7	Equivocal	N/A	N/A	N/A	N/A
46. Single point of access^b^	N/A	N/A	N/A	6 (5 − 7)	53.3	Equivocal	N/A	N/A	N/A	N/A
47. Alignment of regulatory frameworks^b^	N/A	N/A	N/A	7 (7 − 8)	93.3	Appropriate		SI	75	Yes
Functional integration										
48. Human resource management^a^	Equivocal	6.5 (6 − 7)	37.5	6 (4 − 7)	46.7	Equivocal	N/A	N/A	N/A	N/A
49. Information management	Appropriate	8 (7.1 − 9)	81.3	N/A	N/A	Appropriate	FI		62.5	Yes
50. Resource management	Equivocal	6 (5 − 7)	50	5 (3.8 − 7)	40	Equivocal	N/A	N/A	N/A	N/A
51. Support systems and services	Equivocal	5.5 (5 − 6)	68.8	5 (3 − 6)	60	Equivocal	N/A	N/A	N/A	N/A
52. Service management	Appropriate	6 (6 − 7)	43.8	6 (6 − 7)	40	Equivocal		FI	100	Yes
53. Regular feedback of performance indicators^a^	Appropriate	7 (6.1 − 8)	68.8	7 (7 − 9)	86.7	Appropriate		FI	87.5	Yes
Normative integration										
54. Collective attitude	Appropriate	7 (6 − 8)	62.5	7 (6 − 8)	66.7	Equivocal	NI		75	Yes
55. Sense of urgency	Appropriate	7 (6.1 − 8)	68.8	7 (5.8 − 8)	66.7	Equivocal			DA	No
56. Reliable behaviour	Appropriate	7.5 (7 − 8)	81.3	N/A	N/A	Appropriate		NI	62.5	Yes
57. Conflict management	Equivocal	7 (6.1 − 8)	68.8	8 (7 − 8.2)	80	Appropriate		OI	62.5	Yes
58. Visionary leadership^a^	Appropriate	7.5 (6.1 − 8.9)	68.8	9 (8 − 9)	86.7	Appropriate		NI	75	Yes
59. Shared vision	Appropriate	7.5 (7 − 8)	87.5	N/A	N/A	Appropriate	NI		75	Yes
60. Quality features of the informal collaboration	Appropriate	7 (6 − 8)	62.5	7 (6.8 − 8)	73.3	Appropriate			DA	No
61. Linking cultures	Appropriate	7 (7 − 8)	75	N/A	N/A	Appropriate	NI		75	Yes
62. Reputation	Inappropriate	5.5 (5 − 7)	50	6 (3.8 − 7)	40	Equivocal	N/A	N/A	N/A	N/A
63. Transcending domain perceptions	Appropriate	8 (7.1 − 9)	93.8	N/A	N/A	Appropriate			DA	No
64. Trust	Appropriate	8 (8 − 9)	87.5	N/A	N/A	Appropriate		OI	75	Yes

*N/A* not applicable, *DA* disagreement, *CI* clinical integration, *PI* professional integration, *OI* organisational integration, *SI* system integration, *FI* functional integration, *NI* normative integration, *PP* person-focused and population-based care

^#^Results are adapted from Valentijn et al. [6]

^a^Adjustment description Delphi study 1

^aa^Refinement description Delphi study 2

^b^Newly added key features after Round 1 of Delphi study 2

In the second round, the 34 equivocal features were discussed during the face-to-face meeting. This resulted in an extra 17 features rated as appropriate, see Table 3, columns 5–7. The other 17 features remained equivocal after the second round. With regard to the five newly added features, three were rated as appropriate (no. 43, no. 44 and no. 47) and the remaining two (no. 45 and no. 46) as equivocal. To summarise, 45 features were considered appropriate (25 in the first round and 20 in the second round), while 19 features were considered equivocal (see Table 3, column 7).

### Delphi study 2

Fifty features were included in the international Delphi study 2, because they were considered appropriate within the previous national Delphi study (see Table 3, column 2) or international Delphi study 1 (see Table 3, column 7). In the first round of international Delphi study 2, the panel members agreed (≥60 %) on the categorisation of 22 of the 50 features under one of the seven domains of the taxonomy (see Table 3, column 8).

In the second round, the panel members first discussed the descriptions of the seven domains of the taxonomy. The comments on the descriptions of the domains of the taxonomy and changes made in response to them are summarised in Table 4. Subsequently, the panel members discussed the categorisation of the remaining 28 features that were equivocal after the first round. This resulted in an additional 16 features that experts reached an agreement on regarding the categorisation under one of the seven domains of the taxonomy. The remaining 12 (28 minus 16) features were not agreed upon with regard to their categorisation (see Table 3, column 9).

**Table 4:** Statements by experts of international Delphi study 2 on initial descriptions of domains of taxonomy, highlighting main comments and final descriptions

Initial domains and descriptions^#^	Main comments	Adjusted descriptions
1. Clinical integration: The coordination of person-focused care in a single process across time, place and discipline.	• Add that integration is needed for a complex (multi-problem) at stake	1. Clinical or service integration: Coordination of person-focused care for a complex need at stake in a single process across time, place and discipline.
	• Clinical is too strict for the health and social aspects of health (service delivery)	
2. Professional integration: Inter-professional partnerships based on shared competences, roles, responsibilities and accountability to deliver a comprehensive continuum of care to a defined population.	• Add shared understanding among professional groups, since this is of crucial importance for professional integration	2. Professional integration: Inter-professional partnerships based on a shared understanding of competences, roles, responsibilities and accountability to deliver a comprehensive continuum of care to a well-described population.
	• Rephrase defined into well-described population	
3. Organisational integration: Inter-organisational relationships (e.g. contracting, strategic alliances, knowledge networks, mergers), including common governance mechanisms, to deliver comprehensive services to a defined population.	• Use the structure of the description of professional integration to describe organisational integration	3. Organisational integration: Inter-organisational partnerships (e.g. agreements, contracting, strategic alliances, knowledge networks, mergers) based on collaborative accountability and shared governance mechanisms, to deliver a comprehensive continuum of care to a well-described population.
	• The word integration is problematic, as it is the end of the continuum	
	• Add collaborative accountability, since this is essential for organisational integration	
	• Rephrase “well defined” as “well-described”	
4. System integration: A horizontal and vertical integrated system, based on a coherent set of (informal and formal) rules and policies between care providers and external stakeholders for the benefit of people and populations.	• Remove horizontal and vertical integration because it does not clearly describe and is too complex to understand	4. System integration: Coherent set of (informal and formal) political arrangements to facilitate professionals and organisations to deliver a comprehensive continuum of care for the benefit of people and populations.
	• Generally it is difficult to differentiate between organisational and system integration	
	• Add the political influence in the description, since that is the essence of system integration	
	• Also add that system integration has to facilitate the other integration mechanisms such as organisational and professional integration	
5. Functional integration: Key support functions and activities (i.e. financial, management and information systems) structured around the primary process of service delivery, to coordinate and support accountability and decision making between organisations and professionals to add overall value to the system.	• Add that functional integration is the technical enabler for integrated (primary) care	5. Functional integration: Supporting communication mechanisms and tools (i.e. financial, management and information systems) structured around the primary process of service delivery, to provide optimal information as a feedback mechanism for decision support between organisations, professional groups and individuals.
	• Add that communication and feedback mechanism is aimed at facilitating decision making	
6. Normative integration: The development and maintenance of a common frame of reference (i.e. shared mission, vision, values and culture) between organisations, professional groups and individuals.	• Add that normative integration is the cultural enabler for integrated (primary) care	6. Normative integration: Mutually respected cultural frame of reference (i.e. shared mission, vision, values and behaviour) between organisations, professional groups and individuals to achieve shared goals towards person-focused and population based care.
	• Add mutual respect of cultural frame of references	
	• Add that the shared goals should be aimed integrated primary care guiding principles: person-focused and population-based care	
7. Person-focused and population based care: Based on the needs and health characteristics of people and populations care is coordinated across professionals, organisations and support systems.	• Distinguish between the person-focused and population-based domain within the final taxonomy	7. Person-focused and population based care: Based on the needs of people and populations, care is coordinated across professionals, organisations and support systems in order to achieve the triple aim (improving individual experience of care, the health of the population and reducing the costs per capita)
	• Add that the added value is achieving the Triple Aim together	

^#^ Initial domains and descriptions are partially adapted from Valentijn et al. [3], and adjusted descriptions are based on the comments from the expert panel of international Delphi study 2

### Synthesis of results

**Fig. 3:**
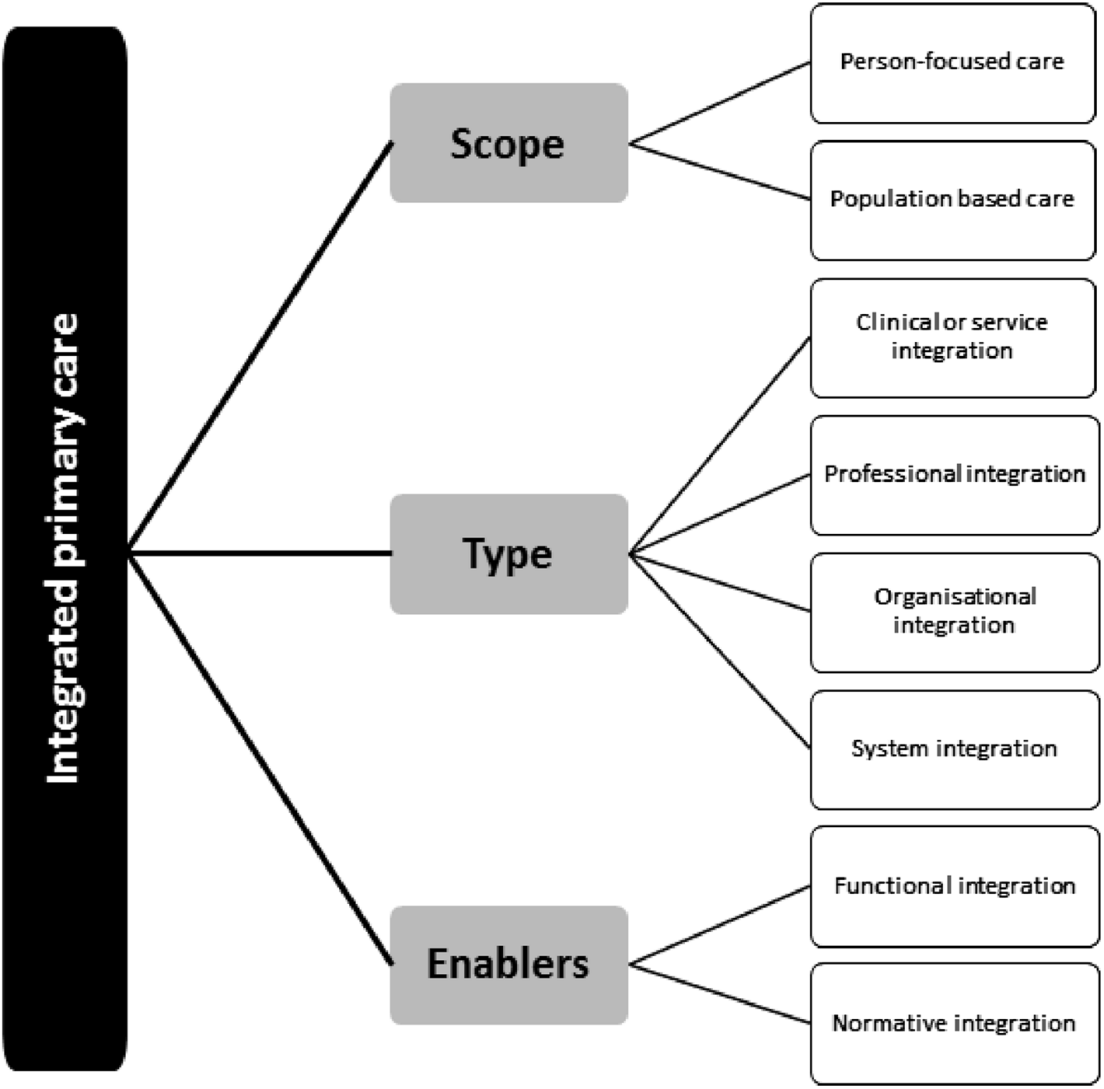
Final taxonomic structure of integrated primary care

During Delphi study 2, it appeared that our final taxonomy to describe an integrated primary care service model could be organised into three main categories: *scope*, *type* and *enablers*. The experts indicated that a distinction should be made between the individual (person-focused care) and population (population-based care) objectives to describe the *scope* of an integrated primary care initiative (see also Table 4). Furthermore, the experts indicated that the clinical, professional, organisational and system domains of our draft taxonomy could be used to describe the various *types* of integration processes. Finally, the qualitative comments of the experts revealed that functional and normative domains of our taxonomy are, respectively, the essential technical and cultural *enablers* for achieving integrated primary care. Therefore, we organised our final taxonomy into these three corresponding categories: 1) scope, 2) type, and 3) enablers of integrate primary care. Based on the comments made during Delphi study 2, the research team split the person-focused and population-based domain, resulting in a total of eight domains. A graphic representation of the final taxonomic structure is presented in Fig. 3.

**Fig. 4:**
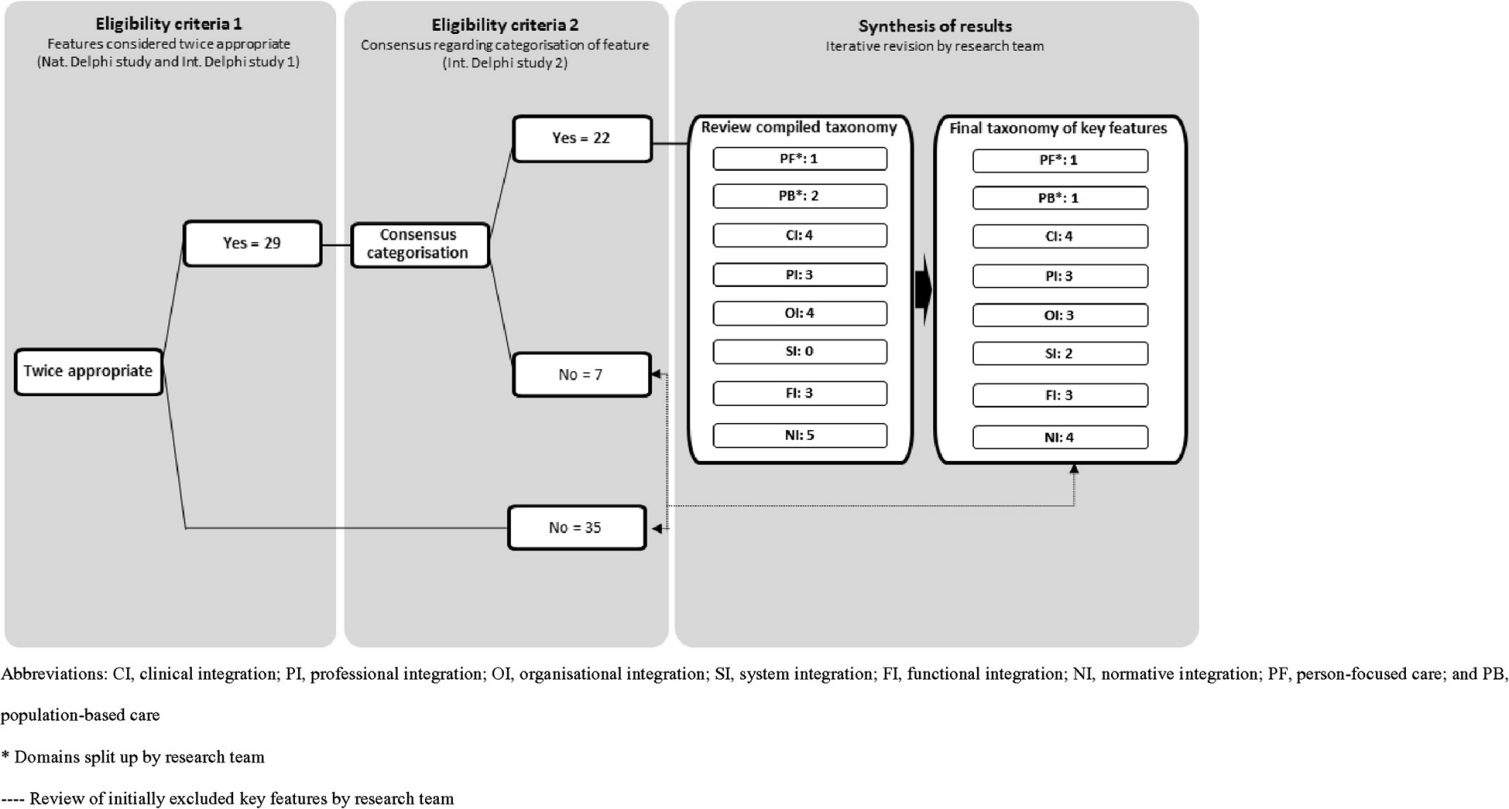
Results of the synthesis process

The final taxonomic structure of eight domains was used to select and categorise the underlying key features. First, the features that were considered appropriate in the previous national Delphi study [6] as well as the current international Delphi study 1 were selected. This resulted in the selection of 29 features (see also Fig. 4). Second, the selected features were categorised within one of the domains of the taxonomy following the results of international Delphi study 2. Twenty-two features could be categorised within one of the eight domains. Third, the compiled taxonomy of 22 key features was reviewed by three authors (PV, MB and IB) on comprehensiveness per domain. The preliminary taxonomy did not contain any features within the system integration domain. A universal judgement was made by the three reviewers to add the features *environmental climate* (Table 3, no. 42) and *alignment of regulatory framework* (Table 3, no. 47) to the system integration domain because the categorisation reached consensus during Delphi study 1 and both features were considered appropriate during Delphi study 2. Finally, the three reviewers decided to merge the *features population needs* (Table 3, no.11) and *population needs as binding agent* (Table 3, no. 29) within the population based domain, *inter-professional governance* (Table 3, no. 17) and *inter-organisational governance* (Table 3, no. 25) within the organisational domain, and *shared vision between professionals* (Table 3, no. 14) and *shared vision* (Table 3, no. 59) within the normative domain due to similar content. The resulting taxonomy of 21 key features is shown in Table 5.

**Table 5:** Final taxonomy of key features

Main categories and domains	Description
**Scope of integrated care**	
*Person-focused care*	
Centrality of client needs	The principle of integrated service delivery is to address the needs of individual clients in terms of medical, psychological and social aspects of health
*Population based care*	
Centrality of population needs ^b^	The principle of integrated service delivery is to address the dominant needs of well-defined populations
**Type of integration processes**	
*Clinical integration*	
Case management	Coordination of care for clients with a high risk profile (e.g. identifying risks, developing policies and guidance)
Continuity	Integrated service delivery aims to provide fluid the processes of care delivery for an individual client
Interaction between professional and client	Attitude and behavioural characteristics between professional and client regarding all health needs of the client
Individual multidisciplinary care plan	Implementation and application of a multidisciplinary care plan at the individual client level
*Professional integration*	
Inter-professional education	Inter-professional education for professionals focused on interdisciplinary service delivery and collaboration
Agreements on interdisciplinary collaboration	Agreements on the establishment of interdisciplinary service delivery and collaboration between the professionals
Value creation for the professional	The value added by the integrated service delivery approach for the individual professional
*Organisational integration*	
Inter-organisational governance ^b^	The governance of the integrated service model is focused on openness, integrity and accountability between the involved organisations and professionals (e.g. joint accountability, appeal on pursued policies and responsibilities)
Inter-organisational strategy	Collective elaborated strategy between the organisations involved in the integrated service model
Trust	The extent to which those involved in the integrated service model trust each other
*System integration*	
Alignment of regulatory frameworks ^a^	Alignment of regulatory frameworks for teamwork, coordination and continuity of care
Environmental climate ^a^	Political, economic and social climate in the environment of the integrated service model (e.g. market characteristics, regulatory framework, and competition)
**Enablers for integration**	
*Functional integration*	
Learning organisations	Collective learning power between the organisations involved in the integrated service model (e.g. joint research and development programs)
Information management	Aligned information management systems within the integrated service model (e.g. monitoring and benchmarking systems)
Regular feedback of performance indicators	Regular feedback of performance indicators for quality improvement and self-reflection
*Normative integration*	
Shared vision ^b^	Collectively shared long-term vision among the people who are involved in the integrated service model
Reliable behaviour	The extent to which the agreements and promises within the integrated service model are fulfilled
Visionary leadership	Leadership based on a vision that inspires and mobilizes people within the integrated service model
Linking cultures	Linking cultures (e.g. values and norms) with different ideological values within the integrated service model

^a^ Features were added at final taxonomy during the review and synthesis process

^b^ Features were merged due to identical or nearly identical content

## Discussion

### Principal findings

This study established an international consensus-based taxonomy to understand the integrated service models that arise in a primary care setting. The national and international Delphi studies resulted in the refinement of our previous taxonomy. The final taxonomy consists of 21 key features of an integrated primary care service model which are distributed over eight integration domains and organised into three main categories: *scope* (person-focused vs. population-based), *type* (clinical, professional, organisational and system) and *enablers* (functional vs. normative). The refinement of the taxonomy is a crucial step towards establishing an instrument that can measure a broad range of integrated service models.

The taxonomy contributes to a deeper understanding of the compound art of integrated care and provides direction for further field testing to identify effective components of integrated service delivery models in a primary care setting. The three main categories of the taxonomy provide a crucial differentiation to clarify and interpret practical examples of integrated care. To begin with, specifying the scope of an integrated care approach as either person-oriented or population-oriented helps to understand and describe the guiding principles and objectives of an integrated care approach. Although person-focused and population-based care could be viewed as opposite approaches, the strength of primary care philosophy is grounded in their symbiosis [2, 11]. Integrated primary care is the crucial point of tangency between public health services, which are more orientated on the population, and medical-oriented services, which are more focused on the individual [1–3]. Consequently, there is a need to specify the balance between person-oriented and population-oriented objectives of an integrated primary care service model. In contrast to more disease specific integrated care models (which are generally more person-focused), the current taxonomy acknowledges that both scopes are needed to improve the provision of continuous, comprehensive and coordinated services in an ambulatory care setting [12].

Second, the original RMIC indicates four equally important *types* of integration processes: clinical, professional, organisational and system integration. However, the results of the Delphi studies indicated that the clinical, professional and organisational integration processes were the most recognised among the experts. This finding corresponds to the fact that these processes have been the prime focus of scientific research and practice [4, 13–15]. The present study also indicates that less emphasis was placed on system integration processes. This result is in contrast with observations in the literature that societal and political influences are essential preconditions for achieving integrated primary care [3, 16, 17]. Research indicates that the development of integrated primary care is more hampered by political influences than technical influences [1]. Furthermore, the Chronic Care Model of Wagner [18] also stresses the importance of embedding integration efforts into the broader societal and political environment. Most of the experts in our study considered organisational integration processes as a systemic whole and found it difficult to differentiate between the system integration types of processes. One possible explanation might be found in the composition of our expert panels, as we did not explicitly include experts with a macro policy background. However, health care practitioners, funders and policymakers have generally a more limited scope compared to the theoretical discourses of integrated care and primary care [19]. The broad inter-sectorial system definitions of both primary care and integrated care have failed to produce practical relevance for practices and policies [6]. Moreover, actors in a public health sector generally have a broader perspective on the social-political aspects of health compared to actors with a healthcare background [20–22]. We believe that more focus should be placed on the system environment when developing and evaluating integrated primary care service models. However, there is still a need to further clarify the domain of system integration and to explore how the different types of integration processes interact. For example, future research might investigate how local regulatory frameworks regarding integrated care influence the organisational and professional integration processes and vice versa.

Finally, the taxonomy assists to clarify and interpret the technical (functional) and cultural (normative) enablers in order to achieve common goals and optimal results. These functional and normative integration conditions seem to be of crucial importance to whether or not clinical, professional, organisational or system integration processes are successfully developed and sustained. Since integrated care spans across many different professional and organisational boundaries and mind-sets, it is crucial to clarify the required functional and normative prerequisites (e.g. data management, feedback, leadership) when developing and evaluating practical examples of integrated primary care.

### Strengths and weaknesses

The strength of this study is the international Delphi study approach to establish a consensus-based taxonomy. The final taxonomy is theoretically grounded on the RMIC [3], has a solid base in available literature [6] and was tested against a wide mix of expert opinions. Nevertheless, the Delphi consensus approach does not necessarily provide the “right” answer to a given problem, but should be viewed as a means to structure group communication and determine the degree of consensus between expert groups [23]. The Delphi approach in this study added substantially to the ongoing debate of defining integrated care.

A potential limitation of this study relates to the selection of experts. We attempted to be inclusive; however, not all experts who were invited were able to participate. Twenty-eight experts refused to participate in Delphi study 2, mainly because the face-to-face meetings were bound to a fixed date and time. We do not expect these rejections to have biased the results substantially because the final panel consisted of a balanced number of experts with both practice and scientific backgrounds. Moreover, the final taxonomy was based on the results from three Delphi studies. Nonetheless, the composition of the expert panels remained biased. The present taxonomy is based on professional (i.e. practical and scientific) values and preferences, while the views of other stakeholder groups (like patients, policymakers or health insurers) are also considered important in integrated care. Different stakeholder groups are likely to have different preferences [24]. This limitation can be solved when the taxonomy is tested in a local setting. All involved stakeholder groups (like patients, professionals, managers, insurers and policymakers) could be asked to comment on the relevance of the features included in the taxonomy and the relative importance per stakeholder could then be adapted accordingly [25–27].

Ultimately, the present taxonomy represents a first step towards a common language for evaluating integrated primary care services. The variety of perspectives of the numerous actors involved in integrated care made us aware of the difficulties of developing a clear, consensus-based, non-overlapping assessment tool and a scoring scale useful for scientific research, policy and practice. Since the taxonomy is grounded on the theoretical concept of integrated primary care, the appropriateness of the taxonomy in other healthcare settings (e.g. hospital settings) should be further explored. Further research should also focus on the development of such a tool with contextualised and non-overlapping items and scoring scales. Finally, the critical challenge is to demonstrate the impact of integrated primary care models in terms of the ‘Triple Aim’ outcomes: 1) improve the individual experience of health care, 2) improve the health of populations, and 3) reduce the per capita costs of care [1]. Therefore, there is a need to further link the performance shaping features of the taxonomy with the three linked outcome measures of the Triple aim to determine the impact and to guide the continues design and redesign of integrated primary care practice.

### Implications for practice and research

The taxonomy is a valuable framework for patient organisations, professionals, managers, commissioners, and policymakers involved in the development of practical examples of integrated primary care. Profiling integrated primary care service models along this taxonomy makes it possible to obtain comprehensive and systematic information, and builds a common knowledge base regarding integrated primary care. Using the taxonomy to compare data across integrated care settings can promote the learning and sharing of (best) practices. Two activities involved in the development of an assessment tool are now pending: firstly, measurement instruments based on the taxonomy to generate reliable and validated quantitative scores; secondly, an agreed upon procedure that measures and incorporates the different perspectives of all the actors involved in integrated care (e.g. patients, professionals, managers, insurers and policymakers).

Once these measurement instruments and procedures are developed, we may be able to understand which interaction patterns achieve better health at a lower cost within a specific context. We plan further work to develop this assessment tool, and invite anyone interested in helping to validate the taxonomy to contact the authors.

## Conclusion

This study established a consensus-based taxonomy for understanding integrated primary care. Based on the theoretical foundations of the RMIC, the final taxonomy now specifies the scope, type and enablers of an integrated primary care service model. This knowledge base provides a crucial differentiation to clarify and support research, policy formulation and implementation regarding the organisation of integrated primary care. For this purpose, the taxonomy has set a developmental agenda for both integrated primary care practice and research.

## Abbreviations

RMIC: Rainbow model of integrated care
UK: United Kingdom
USA: United Stated of America
CI: Clinical integration
PI: Professional integration
OI: Organisational integration
SI: System integration
FI: Functional integration
NI: Normative integration
PF: Person-focused care
PB: Population-based care
